# A pilot study of autologous tumor lysate-loaded dendritic cell vaccination combined with sunitinib for metastatic renal cell carcinoma

**DOI:** 10.1186/s40425-014-0030-4

**Published:** 2014-08-19

**Authors:** Hirokazu Matsushita, Yutaka Enomoto, Haruki Kume, Tohru Nakagawa, Hiroshi Fukuhara, Motofumi Suzuki, Tetsuya Fujimura, Yukio Homma, Kazuhiro Kakimi

**Affiliations:** 1 Department of Immunotherapeutics, The University of Tokyo Hospital, 7-3-1 Hongo, Bunkyo-ku, Tokyo 113-8655, Japan; 2 Department of Urology, The University of Tokyo Hospital, 7-3-1 Hongo, Bunkyo-ku, Tokyo 113-8655, Japan; 3 Department of Urology, Mitsui Memorial Hospital, Izumicho 1, Kanda, Chiyoda-Ku, Tokyo 101-8643, Japan

**Keywords:** RCC, Sunitinib, Dendritic cell, Lysate

## Abstract

**Background:**

Sunitinib, a tyrosine kinase inhibitor currently in use for the treatment of metastatic renal cell carcinoma (mRCC), has been reported to modulate immunosuppressive cells such as myeloid-derived suppressor cells (MDSCs) and regulatory T cells (Tregs) in addition to exerting anti-angiogenic effects. We conducted a clinical trial of dendritic cell (DC)-based immunotherapy together with sunitinib in mRCC patients in an effort to enhance immunotherapeutic efficacy by inhibiting immunosuppressive cells.

**Methods:**

Patients aged ≥20 years with advanced or recurrent mRCC who underwent nephrectomy were eligible for this study. Autologous tumor samples were obtained by surgery under aseptic conditions and used for preparing autologous tumor lysate. About 4 weeks after surgery, leukapheresis was performed to isolate peripheral blood mononuclear cells (PBMCs). DCs were generated from adherent PBMCs in the presence of recombinant human granulocyte macrophage colony-stimulating factor (GM-CSF) (500 IU/ml) and recombinant human IL-4 (500 IU/ml). Autologous tumor lysate was loaded into mature DC by electroporation. Eight patients were enrolled in the study and received sunitinib at a dose of 50 mg p.o. daily for 28 days followed by 14 days of rest. Tumor lysate-loaded DCs were administered subcutaneously every two weeks, with concomitant sunitinib.

**Results:**

No severe adverse events related to vaccination were observed. Sunitinib decreased the frequencies of MDSCs in peripheral blood of 5 patients and of Tregs in 3. Tumor lysate-reactive CD4 or CD8 T cell responses were observed in 5 patients, 4 of whom showed decreased frequencies of Tregs and/or MDSCs. The remaining 3 patients who failed to develop tumor-reactive T cell responses had high levels of IL-8 in their sera and did not show consistent reductions in MDSCs and Tregs.

**Conclusions:**

DC-based immunotherapy combined with sunitinib is safe and feasible for patients with mRCC.

**Trial registration:**

UMIN000002136

## Background

Renal cell carcinoma (RCC) accounts for 2–3% of all adult cancers. Approximately 20–30% of patients present with metastatic disease. Although surgery is the primary curative therapy for localized RCC, the prognosis for patients with advanced metastatic disease is poor, with a 5-year survival rate of <10% [1],[2]. Since the first receptor tyrosine kinase inhibitor (TKI) sorafenib was approved for the treatment of cytokine-refractory metastatic RCC (mRCC), many agents have become available for the treatment of this disease. However, many tumors acquire resistance to these agents by mutating the target genes or activating other pathways that bypass the site of inhibition. This occurs rapidly, often within several months [3]. Therefore, development of other modalities such as immunotherapy is still needed for the treatment of mRCC.

RCC appears to be one of the most immune-sensitive cancers. This has encouraged the use of immunomodulating treatments such as cytokine-based therapy using IL-2 and/or interferon-α (IFN-α) [4],[5]. Nonetheless, nephrectomy is still recommended for patients with mRCC [6], because cytoreductive therapy was shown to provide overall survival benefit in patients treated with IFN-α [7]. Although it is still controversial whether cytoreductive therapy also contributes to the efficacy of TKIs [8], nephrectomy reduces the tumor burden, alleviates symptoms and allows more information on histology to be acquired. In addition, we can utilize the resected tumor as a source of autologous materials, such as tumor lysates, for the production of autologous tumor vaccines. It has been reported that adjuvant treatment with autologous tumor lysate vaccine resulted in a significantly improved overall survival in pT3 stage RCC patients [9]. Antigen-specific vaccination with dendritic cells (DCs) has also been conducted, but with only limited success so far [10]–[15], possibly due to functionally-defective T cell responses in the tumor microenvironment.

It is well accepted that the tumor microenvironment imposes different degrees of immunosuppression allowing the tumor to evade immune responses [16]. These include the delivery of negative costimulatory signals to T cells (via PD-L1, B7-H4) and production of immunosuppressive factors (eg. IL-10, TGF-β, IDO and others). Recently, promising immunotherapeutic strategies have emerged from our understanding of immunoinhibitory pathways termed “immune checkpoints”, which are crucial for maintaining self-tolerance and modulating the duration and magnitude of physiological immune responses. Tumors utilize such immune checkpoints as a resistance mechanism to escape anti-tumor immune responses [17]. Hence, immune checkpoint blockade is a promising approach to activating antitumor immunity. The antibodies that block CTLA-4- and PD-1-dependent interactions have been successfully applied for the treatment of mRCC [18]–[21].

In addition, different regulatory cell populations, such as MDSCs or Tregs, are involved in this process. The accumulation of MDSCs as well as the suppression of T-cell function in mRCC patients has been reported [22],[23]. TKIs such as sunitinib and sorafenib were approved some time ago and are now the mainstay for the treatment of mRCC [24]–[26]. In addition to its anti-angiogenic effects, sunitinib has been demonstrated to modulate immunosuppressive MDSCs in human [27] and mouse [28]. It has also been reported that sunitinib reverses type-1 immune suppression and decreases Tregs in renal cell carcinoma patients [29]. Furthermore, sunitinib, unlike sorafenib, does not inhibit specific T cell responses [30]. Therefore, sunitinib appears to be a promising molecular target drug for combination therapy together with cancer vaccines for mRCC.

Here, we report the results of a clinical trial in which we evaluated the safety and feasibility of DC-based vaccination combined with sunitinib for mRCC patients and tested whether sunitinib enhances immune responses by reducing immunosuppressive cells.

## Results

### Patients

Eight patients (5 men and 3 women) with a median age of 68 yr (range, 55–75) were enrolled in this study (Table 1). Two patients were categorized into the MSKCC poor risk group and the other six as having an intermediate risk. One patient (#1808) had unclassified RCC, while the other seven had clear cell RCC. Two patients, #1802 and #1803, received sunitinib or IFN-α and radiation for bone metastasis, respectively, before surgery.

**Table 1:** Patients’ characteristics

**Patient ID**	**Age/Sex**	**Stage**	**Meta site**	**MSKCC**	**Histology**	**Grade**	**Prior treatment**
1802	72/F	pT3aN2M1	Lung, LN	Poor	Clear cell	2 > 3	Sunitinib
1803	72/M	pT3bN0M1	Liver, lung, bone	Poor	Clear cell	3 > 2	IFN-α, radiation
1806	72/F	pT4N1M1	Lung, LN	Intermediate	Clear cell	3	no
1808	75/M	pT3aN2M1	Lung, LN, bone	Intermediate	Unclassified	3	no
1812	61/M	pT1bN1M1	LN	Intermediate	Clear cell	2 > 1> > 3	no
1814	55/M	pT3aN0M1	Lung	Intermediate	Clear cell	2	no
1817	64/F	pT3bN1M1	Lung, LN, bone	Intermediate	Clear cell	3 > 2	no
1823	57/M	pT1N0M1	Lung, pleura	Intermediate	Clear cell	2 > 3	no

MSKCC**,** Memorial Sloan Kettering Cancer Center risk criteria; LN, lymph node.

### DC Vaccine combined with sunitinib

**Figure 1:**
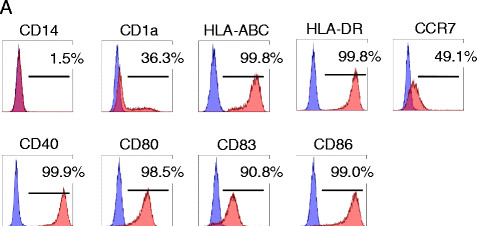
Surface phenotype of DCs; specific mAb staining (red) and isotype control mAb staining (blue).

DCs were successfully generated from all 8 patients (Table 2). Final concentrations of tumor lysate per 10^7^ DCs ranged from 0.44 to 1.33 mg (mean value, 0.90 mg). Flow cytometric analysis of the harvested tumor lysate-loaded DCs revealed a phenotype characteristic of mature DCs with high expression of CD40, CD80, CD83, CD86, HLA-ABC, HLA-DR, and CCR7 (Figure 1 and Table 3). While there were some differences in the fluorescent intensities of these molecules among patients’ DCs (Additional file 1), the phenotype of these DCs were quite comparable. None of the DC preparations was microbially contaminated. Each patient was given 1×10^7^ DCs at each time point, with the exception of one patient (#1823) who received 0.5×10^7^ DCs (Table 2). Patients received 6 vaccinations and sunitinib at a dose of 50 mg p.o. daily for 28 days followed by 14 days of rest, according to the schedule (Additional file 2). Vaccination was well-tolerated and no severe vaccination-related toxicity or autoimmune manifestations were observed in any patient.

**Table 2:** Quality and quantity of tumor lysate-loaded DCs

**Patient ID**	**Tumor lysate used for EP (mg)**	**DCs used for EP (x10**^**7**^**)**	**Tumor lysate (mg)/ 10**^**7**^**DCs**	**Number of DCs injected**	**Viability (%)**
1802	15	29.5	0.51	1x10^7^	82.9
1803	15	18.3	0.82	1x10^7^	82.1
1806	20	17.1	1.17	1x10^7^	92.8
1808	7	16.0	0.44	1x10^7^	83.6
1812	20	20.1	1.00	1x10^7^	89.0
1814	20	21.5	0.93	1x10^7^	91.7
1817	20	20.0	1.00	1x10^7^	87.5
1823	20	15.0	1.33	0.5 x10^7^	92.4

EP, electroporation.

**Table 3:** The surface phenotype of DCs

**Patient ID**	**% Expression**								
	CD14	CD1a	HLA-ABC	HLA-DR	CCR7	CD40	CD80	CD83	CD86
1802	7.8	51.6	98.7	99.7	10.6	99.9	96.4	60.1	96.3
1803	1.4	79.6	99.2	99.9	38.7	99.4	99.1	94.1	99.5
1806	1.5	36.3	99.8	99.8	49.1	99.9	98.5	90.8	99
1808	5.2	53.3	98.8	99.5	43.1	99.4	96.7	75.6	98.7
1812	0.6	83	99.9	99.7	10.3	99.8	99.2	87.4	97.9
1814	0.6	66.5	99.4	99.4	58.3	99.6	98.7	91.5	99.2
1817	1.4	34.7	99.7	98.3	50.3	99.8	96.7	61.8	98
1823	0.8	50.4	99.7	99.6	30.5	99.7	99.3	95.4	99.3

### Frequencies of MDSCs and Tregs in peripheral blood

**Figure 2:**
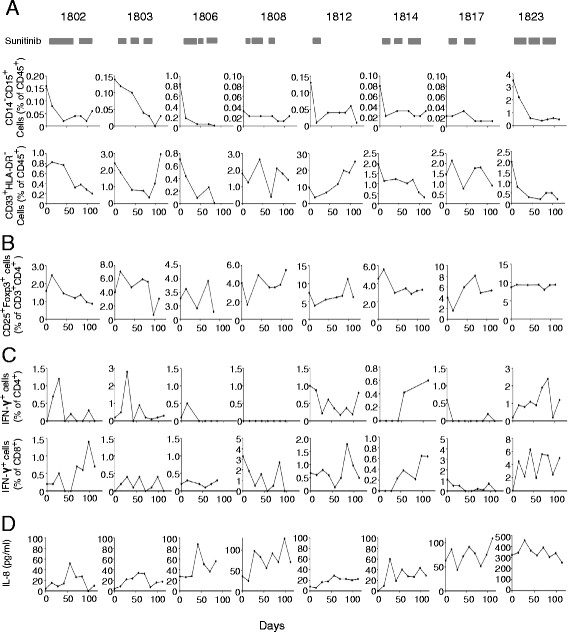
**Immunomonitoring. A**. Percentages of MDSCs by two criteria. **B**. Percentages of Tregs. **C**. Changes of tumor-reactive IFN-γ^+^ cells (% of CD4^+^ or CD8^+^ T cells). Assay was performed as described in Methods section. **D**. The concentration of IL-8 in sera measured by a cytofluorometry-based ELISA system at different time points during treatment of the 8 patients.

MDSCs in peripheral blood were evaluated by two criteria (percent of CD14^−^CD15^+^ or CD33^+^HLA-DR^−^ cells within the Dye780^−^CD45^+^ population) (Additional file 3). In individual patients, decreased percentages of MDSCs were observed in 5 of the 8 patients (#1802, #1803, #1806, #1814, and #1823) by both criteria (Figure 2A and Table 4) compared to pretreatment baseline. No marked changes were observed in patients #1808, #1812 and #1817. Sunitinib significantly reduced the average percentage of CD14^−^ CD15^+^ MDSCs in 8 patients from 0.62 ± 1.20% (mean ± SD) at the baseline to 0.083 ± 0.17% at the 6th DC injection (*p* = 0.0039, Wilcoxon signed-rank test); the average percentage of CD33^+^HLA-DR^−^ MDSCs in 8 patients did not change (2.57 ± 2.86% at the baseline and 3.17 ± 6.73% after sunitinib treatment) (*p* = 0.23, Wilcoxon signed-rank test). For Tregs, the percentages of CD25^+^Foxp3^+^ cells within the Dye450^−^CD3^+^CD4^+^ population (Additional file 3) were found to be decreased relative to the baseline in patients #1802, #1803 and #1814, but not in patients #1806, #1808, #1812, #1817 and #1823 (Figure 2B). However, there was no statistical difference (*p* = 0.273, Wilcoxon signed-rank test).

**Table 4:** Immune responses and clinical outcomes in 8 patients

**ID**	**No. DC injection**	**DTH**	**CD4 T cell response**	**CD8 T cell response**	MDSCs*	Tregs*	**IL-8**^**†**^	**Change in Target Lesions (%)**	**Clinical Response**^**‡**^	**PFS**^**§**^**(d)**	**OS**^**§**^**(d)**	**Prognosis**
1802	6	+	+	+	decreased	decreased	low	−25.4	SD	173	339	Dead
1803	6	-	+	-	decreased	decreased	low	0	SD	200	353	Dead
1806	6	-	-	-	decreased	no change	high	−18.4	N.A.^¶^	100	100	Dead
1808	6	-	-	-	no change	increased	high	−5.4	SD	155	193	Dead
1812	6	-	-	+	no change	no change	low	30.3	PD^#^	101	1140^||^	Alive
1814	12	+	+	+	decreased	decreased	low	−100	CR	347	1127^||^	Alive
1817	6	-	-	-	no change	increased	high	−27.8	PD^**^	88	206	Dead
1823	12	+	+	+	decreased	no change	high	−35.3	PR	342^||^	342^||^	Alive

PFS, progression free survival; OS, overall survival; CR, complete response; PR, partial response; SD, stable disease; PD, progressive disease.

*Compared to the baseline.

^†^High or low is defined as more or less than 60 pg/ml in sera.

^‡^4wks after last injection.

^§^From the registration (days).

^¶^Withdrawn from the study by sudden hypertensive cerebral hemorrhage.

^||^A censored case due to the termination of the study.

^#^After surgical removal of target lesion (LN metastasis), no recurrence was observed.

^**^Though target lesion became smaller, accumulation of pleural effusion was increased.

### DTH reactions and tumor-reactive T cell responses

DTH testing was performed in all 8 patients to detect tumor lysate-reactive responses. Three patients (#1802, #1814 and #1823) had positive DTH reactions (Table 4). Tumor lysate-reactive CD4^+^ and CD8^+^ T cell responses in all patients were further investigated in vitro using the IFN-γ secretion assay at different time points after vaccination. Data from an individual patient #1802 are shown in Additional file 3. Before vaccination, the percentage of CD8^+^ IFN-γ^+^ T cells after simulation with EP-DCs or unloaded DCs was essentially identical (1.6%-vs-1.4%, respectively). However, after vaccination, a higher percentage of CD8^+^ IFN-γ^+^ T cells was observed on stimulation with EP-DCs (2.9%) than with unloaded DCs (1.5%). Similarly, a higher percentage of CD4^+^ IFN-γ^+^ T cells was observed on stimulation with EP-DCs (4.5%) than with unloaded DCs (3.0%). These T cell responses fluctuated during the course of treatment and no statistically significant difference in the increase of IFN-γ^+^ T cells after vaccination was detected. Figure 2C shows the percentage of tumor lysate-reactive IFN-γ^+^ cells (both CD4^+^ as well as CD8^+^ T cells) for all 8 patients. When the percentages at any point after vaccination are elevated 3-fold higher than those at the baseline (mean value of the percentages at days 0 and 14), the tumor-reactive T cell responses are considered to be positive. By this criteria, the induction of tumor lysate-reactive CD4^+^ T cell responses were detected in patients #1802, #1803, #1814 and #1823; patients #1802, #1812, #1814 and #1823 had tumor-reactive CD8^+^ T cell responses (Table 4). The T cell responses were detected even at the time of registration in Patients #1808 and 1812.

### Concentration of IL-8 in the sera

To search for biomarkers predicting responsiveness to combination therapy with sunitinib and DC-based immunotherapy, we analyzed concentrations of IFN-*γ*, IL-1*β*, IL-2, IL-4, IL-5, IL-6, IL-8, IL-10, IL-12 p70, TNF-*α*, and TNF-*β* in sera from the 8 patients before and during treatment. With the exception of IL-8, which was present at different levels in all patients, serum cytokines were barely detectable. Patients #1806, #1808, #1817 and #1823 had greatly elevated levels of >60 pg/ml IL-8 during treatment (Figure 2D and Table 4), whereas patients #1802, #1803, #1812, and #1814 had basal levels <60 pg/ml.

### Clinical responses

The follow-up period ranged from 100 to 1140 days (Table 4). Except for one patient who died of a brain hemorrhage due to hypertension, patients remained alive during the trial with a median overall survival (OS) of 346 days and median progression-free survival (PFS) of 164 days. One patient achieved a complete response (CR), another patient had a partial response (PR), 3 had stable disease (SD) and 2 had progressive disease (PD) according to the RECIST criteria (Table 4). Patient #1814 who achieved the CR was one of three patients who had developed DTH, as well as CD4^+^ and CD8^+^ T cell responses. In this patient, the percentages of both MDSCs and Tregs decreased during treatment. In the CT scan, the size of the mass in the left lung decreased from 17.9 mm to 8.2 mm in diameter after 6 immunizations and had disappeared after 10 (Additional file 4). The other patient who had a DTH reaction, #1802, also had CD4^+^ and CD8^+^ T cell responses, as well as decreased MDSCs and Tregs, and low IL-8. She manifested SD in spite of multiple tumor metastases in the lung (Additional file 4). Her quality of life was markedly improved by a reduction of the pleural effusion (Additional file 4). As shown in Additional file 4, the tumor volume was decreased and pleural effusion was reduced in patient #1823, who develop also positive DTH, CD4^+^ and CD8^+^ T cell responses (Table 4). Patient #1812 was defined as PD when target lesion, supraclavicular lymph node metastasis, was enlarged by 30.3% in size. Therefore, he received surgery to resect the metastatic lymph node and no recurrence was observed with no further treatment.

### Safety

The most common adverse events were hand-foot syndrome, stomatitis, peripheral edema and other skin disorders (Table 5). Sunitinib-related severe adverse events were hypertension and hematological and laboratory abnormalities. They were managed with interruption of sunitinib and were reversible in most cases, except for a fatal hypertensive intracranial hemorrhage in patient #1806 who had no brain metastasis. No severe adverse events related to DC therapy were observed.

**Table 5:** Adverse Events and Laboratory abnormalities

	**Grade**					
Adverse Events, Regardless of Causality	All	1	2	3	4	5
*General disorders*						
Fatigue	2		2			
Pyrexia	2	1	1			
Insomnia	1		1			
*Gastrointestinal disorders*						
Dyspepsia	2		2			
Dysgeusia	2	1	1			
Diarrhea	2	2				
Nausea	1	1				
Esophagitis	1	1				
*Respiratory, thoracic and mediastinal disorders*						
Cough	1		1			
*Musculoskeletal and connective tissue disorders*						
Back pain	3	1	2			
*Metabolism and nutrition disorders*						
Hypothyroidism	4		4			
*Skin and subcutaneous tissue disorders*						
Hand-foot syndrome	8	2	6			
Stomatitis	4	2	2			
Peripheral Edema	4	3	1			
Anal diseases	3		3			
Skin ulceration	1		1			
Pruritus	1		1			
Trichophytosis	1		1			
Rash	1	1				
*Vascular disorders*						
Hypertension	3		1	1		1*
*Hematological and other laboratory abnormalities*						
Anemia	3		1	2		
Leukopenia	3			3		
Neutropenia	3			3		
Lymphocytopenia	3			3		
Thrombocytopenia	3			3		
Increased creatinine	2			2		

*Intracranial hemorrhage.

## Discussion

Here we report a clinical trial of DC-based immunotherapy combined with sunitinib in mRCC patients. We evaluated the safety and feasibility of this approach. In the course of treatment, one patient developed cerebral hemorrhage due to hypertension. However, no severe vaccination-related toxicity or autoimmunity was observed in any of the 8 patients treated. Sunitinib decreased the frequencies of peripheral blood MDSCs and/or Tregs. Vaccination with tumor lysate-loaded DCs induced tumor-reactive CD4^+^ and/or CD8^+^ T cell responses. The treatment showed some clinical benefits in patients possibly linked to successful control of immunosuppressive cells and induction of T cell responses. This was particularly notable in patient #1814 where lung metastases disappeared. However, there is a possibility that these clinical responses are solely due to sunitinib rather than vaccine-induced immune response, since the DC was given concurrently with sunitinib which is an active drug for the treatment of RCC.

Consistent with previous reports [27],[29], we observed reduced percentages of MDSCs during sunitinib treatment, but only in 5 of 8 patients (Figure 2A and Table 4). Of these 5, 4 developed increased tumor-reactive T cell responses. However, the very low number of patients included in this study and the fluctuations in magnitude of T cell responses during the course of treatment make it difficult to conclude the relationship between MDSC and T cell responses. Regarding mechanisms underlying the modulation of MDSCs by sunitinib, it has been shown that this agent inhibits STAT3 signaling. This induces apoptosis in murine MDSCs, where STAT3 is a critical factor responsible for their expansion [31],[32]. On the other hand, GM-CSF accumulating in the tumor expands MDSCs to promote sunitinib-resistance due to preferential STAT5 activation, which cannot be suppressed by sunitinib [33]. Thus, to understand the different sensitivity of MDSCs to sunitinib in different mRCC patients, the STAT3 or STAT5 activation status in the MDSCs and expression of cytokines such as GM-CSF in the tumor would need to be investigated.

A decreased percentage of Tregs after sunitinib treatment was also observed, although only in 3 of the 8 patients (Figure 2B and Table 4). The mechanism underlying regulation of Tregs by sunitinib remains unclear. It has been proposed that the reduction of Tregs by sunitinib may be an indirect effect of the downregulation of MDSCs and/or increases in IFN-γ production [27]. In our case, reduced frequencies of Tregs were observed in 3 of the 5 patients who did show reduced MDSCs. No reduction of Tregs was seen in a further 3 of 3 patients in whom there was no reduction of MDSCs. Nevertheless, the number of patients was too small to lead to any conclusion.

To identify biomarkers for predicting outcome of combination sunitinib and DC-based immunotherapy, we tested a wide range of cytokines (IFN-*γ*, IL-1*β*, IL-2, IL-4, IL-5, IL-6, IL-8, IL-10, IL-12 p70, TNF-*α*, and TNF-*β*) in sera from patients before and during treatment. We found IL-8 in all patients, with 4 having highly elevated levels (>60 pg/ml) during treatment. IL-8 is a member of the CXC family of chemokines and is a potent proangiogenic factor [34]. Renal cell carcinoma has been shown to produce IL-8, and IL-8 expression is known to cause mRCC resistance to sunitinib [35],[36]. IL-8 angiogenic signaling is thought to functionally compensate for the inhibition of VEGF/VEGFR-mediated angiogenesis. Further, the secretion of IL-8 from cancer cells may have a variety of effects on the tumor microenvironment, because the IL-8 receptors CXCR1 and CXCR2 are expressed on cancer cells, endothelial cells, neutrophils and tumor-associated macrophages. It has been shown that production of IL-8 by tumors induces Treg migration into tumors [36]. IL-8 produced by tumor cells may also recruit MDSCs into tumor sites. Therefore, high IL-8 expression may contribute to shaping the immunosuppressive environment in the tumor and inhibiting tumor-reactive T cell responses. In this study, no reduction of IL-8 was achieved by sunitinib (Figure 2D). Therefore, targeting IL-8 signaling may be required for improving this cancer vaccine.

Cancer immunotherapy based on the regulation of immunosuppressive cells, soluble factors, and signaling pathways are now considered essential element of the treatment of cancer [37]. Similar effects are also achieved by molecular targeted therapy, which primarily aims to inhibit molecular pathways that are crucial for tumor cell growth and survival. Importantly, such small molecule inhibitors may also modulate the immune system, which raises the possibility that targeted therapy might be effectively combined with immunotherapy to improve clinical outcomes [38]. This may indeed be the case in our small pilot study. A reduction of immunosuppressive cells by sunitinib likely contributed to stimulating anti-tumor immune responses induced by tumor lysate-loaded DC vaccines.

Initially 15 patients were planned to be included in this study; we terminate the study with 8 patients reproted here, because other TKIs, pazopanib and axitinib, and mTOR inhibitors, temsirolimus and everolimus, are now available for the RCC treatment in addition to sunitinib and sorafenib. A new pilot study is currently underway to determine the better combination of these molecular target drugs with DC-based immunotherapy. Though our study has some limitations in that this is a single institution study and sample size was only 8 patients, our results support the notion that immunotargeted therapy represents an appropriate future direction for developing successful treatment of mRCC.

## Conclusions

This pilot study of DC-based therapy together with sunitinib for mRCC patients has documented the safety and feasibility of this approach. The reduction of both MDSCs and Tregs was achieved by sunitinib in patients whose serum IL-8 levels were not excessive. Autologous tumor lysate-loaded DCs in combination with sunitinib induced both CD4^+^ and CD8^+^ T cell responses in mRCC patients.

## Methods

### Patient selection

A pilot study of DC-based immunotherapy combined with sunitinib in mRCC patients was conducted. The primary endpoints were the safety and feasibility of this approach; the secondary endpoints were to obtain immunological proof of concept and preliminary data for anti-tumor effect, overall survival (OS) and progression-free survival (PFS). Patients aged ≥20 years with advanced or recurrent mRCC who underwent nephrectomy were eligible for this clinical study of DC therapy combined with sunitinib. To be included, patients had to have an Eastern Cooperative Oncology Group performance status (PS) of 0, 1 or 2, normal kidney, liver, and bone marrow function, and at least 1 measurable cancer lesion assessed by computed tomography. Patients positive for anti-adult T-cell leukemia-associated antigen or anti-human immunodeficiency virus antibody, other primary cancers, uncontrolled infection, active enterocolitis, severe heart disease, severe drug allergy, cryoglobulinemia, or autoimmune disease, were excluded from the study. Those receiving systemic steroid therapy, who were pregnant or lactating, or who had brain metastasis and hypertension were also excluded. The research protocol was approved by the Ethical Committee of our institution and was registered at the University Hospital Medical Information Network Clinical Trials Registry (UMIN-CTR) (Unique trial number: UMIN000002136) on July 2, 2009. Written informed consent was obtained from each patient before they entered the study. The study was performed in accordance with the Declaration of Helsinki.

### Generation of DCs

About 4 weeks after surgery, patients underwent leukapheresis to isolate peripheral blood mononuclear cells (PBMCs) using a Fresenius AS.TEC204 with the C4Y white blood cell set. Approximately 5 ×10^9^ PBMCs from each patient were allowed to adhere to tissue culture flasks in AIM-V medium (Invitrogen, Carlsbad, CA) at 37°C. After one hour, nonadherent cells were removed by washing with warm medium. To generate immature DCs, adherent PBMCs were cultured in AIM-V for 5 days in the presence of recombinant human granulocyte macrophage colony-stimulating factor (GM-CSF) (500 IU/ml; Berlex Laboratories, Montville, NJ) and recombinant human IL-4 (500 IU/ml; CellGenix Technologie Transfer GmbH, Freiburg, Germany). Immature DCs were then matured by adding GM-CSF (250 IU/ml), recombinant human IL-4 (250 IU/ml), tumor necrosis factor (TNF-α) (0.01 μg/ml; CellGenix Technologie Transfer GmbH), prostaglandin E2 (PGE2) (1 μg/ml; Sigma, St. Louis, MO) and zoledronate (5 μM; Novartis, Basel, Switzerland) for a further 2 days [39].

### Preparation of tumor lysates and electroloading of dendritic cells

Autologous tumor samples were obtained by surgery under aseptic conditions. Tumor tissues were minced with a scalpel in phosphate-buffered saline (PBS). The samples were then lysed by six freezing and thawing cycles, sonicated and centrifuged to produce tumor lysate. Finally the supernatant was filtered using 0.22-μm pore-size filters. The quantitation of total protein was performed using BCA Protein Assay Kit (Pierece Biotechnology, Rockford. IL, USA) according to the manufacturer’s instruction. Colorimetric changes were detected by VersaMax microplate reader (Molecular Device Japan, Tokyo, JAPAN) at the wavelength of 562 nm with Softmax Pro software (Molecular Device Japan). Autologous tumor lysate was loaded into mature DCs using a MaxCyte GT electroporation-based system (MaxCyte Inc, Gaithersburg, MD) according to the manufacturer’s instructions [40]. Tumor lysate-electroporated DCs, designated EP-DCs, were cryopreserved with 1 ml of autologous serum containing 10% DMSO and stored in liquid N_2_ until use.

### Immunization schedule

After leukapheresis, patients received sunitinib at a dose of 50 mg p.o. daily for 28 days followed by 14 days of rest. Two weeks after leukapheresis, patients received 1x10^7^ EP-DCs subcutaneously in the deltoid region; DC injection was repeated biweekly six times in total, extended to 12 for one long-surviving patient. For immunomonitoring, peripheral blood was drawn before DC therapy, at each treatment time point and 4 weeks after the last treatment. PBMCs were isolated by density gradient centrifugation using Lymphoprep (Axis-Shield, Oslo, Norway) and stored in liquid N_2_ until use. Adverse events were graded according to National Cancer Institute-Common Terminology Criteria for Adverse Events version 4.0. Clinical responses were assessed by computed tomography and classified as complete response (CR), partial response (PR), stable disease (SD), or progressive disease (PD) according to the Response Evaluation Criteria in Solid Tumors (RECIST) criteria, version 1.1 [41].

### IFN-γ secretion assay

PBMCs (1×10^6^) from each time point and EP-DCs (1×10^5^) were thawed and resuspended in AIM-V medium supplemented with 10% heat-inactivated pooled human serum (complete medium), and co-cultured in a 24-well plate at 37°C in a 5% CO_2_ atmosphere for 2 days. Recombinant human IL-2 (Chiron, Emeryville, CA) was then added every 2–3 days to a final concentration of 50 IU/ml for another 12 days. The cultured PBMCs were harvested and used as responder cells, as described below. The IFN-γ secretion assay was carried out according to the manufacturer’s protocol (Miltenyi Biotec, Bergisch Gladbach, Germany) [42]. Briefly, 1 × 10^6^ responder cells were stimulated with 1 × 10^5^ EP-DCs or mature DCs without electroporation (unloaded DCs) in complete medium for 4 hr at 37°C in a 5% CO_2_ atmosphere. The cells were then washed and suspended in 100 μl of cold PBS, and treated with a mouse anti-IFN-γ antibody (IFN-γ catch reagent) (2 μl) for 5 min on ice. The cells were then diluted in complete medium (1 ml) and placed on a slowly rotating device (Miltenyi Biotec) to allow IFN-γ secretion at 37°C in a 5% CO_2_ atmosphere. After incubation for 45 min, the cells were washed with cold PBS and treated with Fixable viability dye eFluor 450 (eBioscience, San Diego, CA), PE-labeled anti-IFN-γ (detection reagent), Alexa Fluor 647-labeled anti-human CD3 (Biolegend, San Diego, CA), PC5-labeled anti-human CD8 (Beckman Coulter, Fullerton, CA), and PECy7-labeled anti-human CD4 (Biolegend) mAbs. After incubation for 10 min at 4°C, the cells were washed and analyzed on a Gallios Flow Cytometer (Beckman Coulter).

### Tregs and MDSCs

Analysis of Treg percentages in patient PBMC was carried out on thawed samples. Cells were stained in fluorescence-activated cell sorting (FACS) buffer (1× PBS with 2% heat-inactivated fetal bovine serum and 0.02% sodium azide). Nonspecific antibody binding was blocked by pretreatment with Clear Back (Human Fc receptor blocking reagent, MBL, Nagoya, Japan). Cells were stained with Dye450, Alexa Fluor 647-labeled anti-CD3, Alexa Fluor 488-labeled Foxp3, PE-Cy5-labeled CD4, and PE-labeled CD25 Abs according to the instructions for use of the Human Treg Flow Kit (Biolegend). MDSCs were also analyzed by FACS on thawed patient PBMC stained with Dye780, ECD-labeled CD14 (Beckman Coulter), FITC-labeled CD15 (Biolegend), PE-Cy5-labeled CD33 (Biolegend), and PE-labeled HLA-DR (BD Biosciences) Abs for 30 min at 4°C. Cells were washed in buffer and then fixed in 1% paraformaldehyde and analyzed by flow cytometry.

### Delayed-type hypersensitivity (DTH)

EP-DCs or unloaded DCs were injected intradermally into different forearms. DTH reactions were evaluated 24 and 48 hours after the 6^th^ injection of DCs and considered to be positive when a skin reaction (>10 mm diameter of erythema) was triggered by EP-DCs but not unloaded DCs.

### TH1/TH2 cytokine quantification

Amounts of IFN-*γ*, IL-1*β*, IL-2, IL-4, IL-5, IL-6, IL-8, IL-10, IL-12 p70, TNF-*α*, and TNF-*β* in patients′ sera were quantified by a cytofluorometry-based ELISA system (Flowcytomix, Bender Medsystems GmbH, Austria). Standard curves for each cytokine were generated using the reference cytokine concentrations supplied by the manufacturer. Cytokines in sera from patients at different time points were estimated according to the manufacturer’s instructions. Raw data of the FC bead assay were analyzed by FlowCytomixPro2.3 software.

### Statistical analysis

The statistical analyses of immunological parameters and prognostic factors (PFS or OS) were performed using Wilcoxon signed-rank test and Kaplan-Meier method, respectively, with JMP software, version 9.0.3 (SAS Institute Inc., Cary, NC, USA).

## Abbreviations

RCC: Renal cell carcinoma

mRCC: Metastatic RCC

TKI: Tyrosine kinase inhibitor

MDSCs: Myeloid-derived suppressor cells

Tregs: Regulatory T cells

PBMCs: Peripheral blood mononuclear cells

GM-CSF: Granulocyte macrophage colony-stimulating factor

DCs: Dendritic cells

TNF-α: Tumor necrosis factor α

PGE2: Prostaglandin E2

OS: Overall survival

PFS: Progression-free survival

CR: Complete response

PR: Partial response

SD: Stable disease

PD: Progressive disease

RECIST: Response Evaluation Criteria in Solid Tumors

PBS: Phosphate-buffered saline

## Competing interests

Department of Immunotherapeutics is an endowed department supported by financial contributions from Medinet Co. Ltd. (Yokohama, Japan). Dr. Kazuhiro Kakimi received research support from Medinet Co. Ltd. The costs of the entire DC culture production and part of the immunological assays were covered by Medinet Co. Ltd. The study sponsors had no involvement in study design; collection, analysis, and interpretation of data; writing the report; and the decision to submit the report for publication. No potential conflicts of interest were disclosed by the other authors.

## Authors’ contributions

Conceived and designed the study: HM, YE, YH and KK. Performed the clinical study: HM, YE, HK, TN, HF, MS, TH, and KK. Analyzed the data: HM and KK. Wrote the paper: HM, YE, YH and KK. All authors read and approved the final manuscript.

## Additional files

## Supplementary Material

Additional file 1: **The mean fluorescent intensity (MFI) of the surface expression of immunological molecules.** Tabular data. Click here for file

Additional file 2: **Schedule for DC vaccination combined with sunitinib in this clinical trial.** Supplementary figure. Click here for file

Additional file 3: **Data from an individual patient.** Supplementary figure. Click here for file

Additional file 4: **Computed tomography (CT) images.** Supplementary figure. Click here for file
