# Supplementary figures and images for: A pilot study of autologous tumor lysate-loaded dendritic cell vaccination combined with sunitinib for metastatic renal cell carcinoma
Source: J Immunother Cancer. 2014 Aug 19;2:30. doi: 10.1186/s40425-014-0030-4 (PMC4331924; doi:10.1186/s40425-014-0030-4)

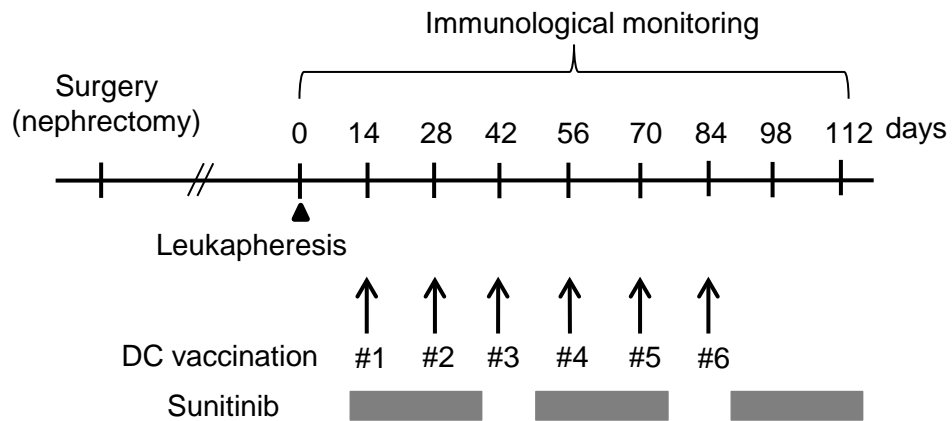

**Additional File 2.** Schedule for DC vaccination combined with sunitinib in this clinical trial.

Supplement: Additional file 2: — Schedule for DC vaccination combined with sunitinib in this clinical trial. Supplementary figure. [file s40425-014-0030-4-S2.pdf]
