# Supplementary material for: A pilot study of autologous tumor lysate-loaded dendritic cell vaccination combined with sunitinib for metastatic renal cell carcinoma
Source: J Immunother Cancer. 2014 Aug 19;2:30. doi: 10.1186/s40425-014-0030-4 (PMC4331924; doi:10.1186/s40425-014-0030-4)
Supplement: Additional file 3: — Data from an individual patient. Supplementary figure. [file s40425-014-0030-4-S3.pdf]

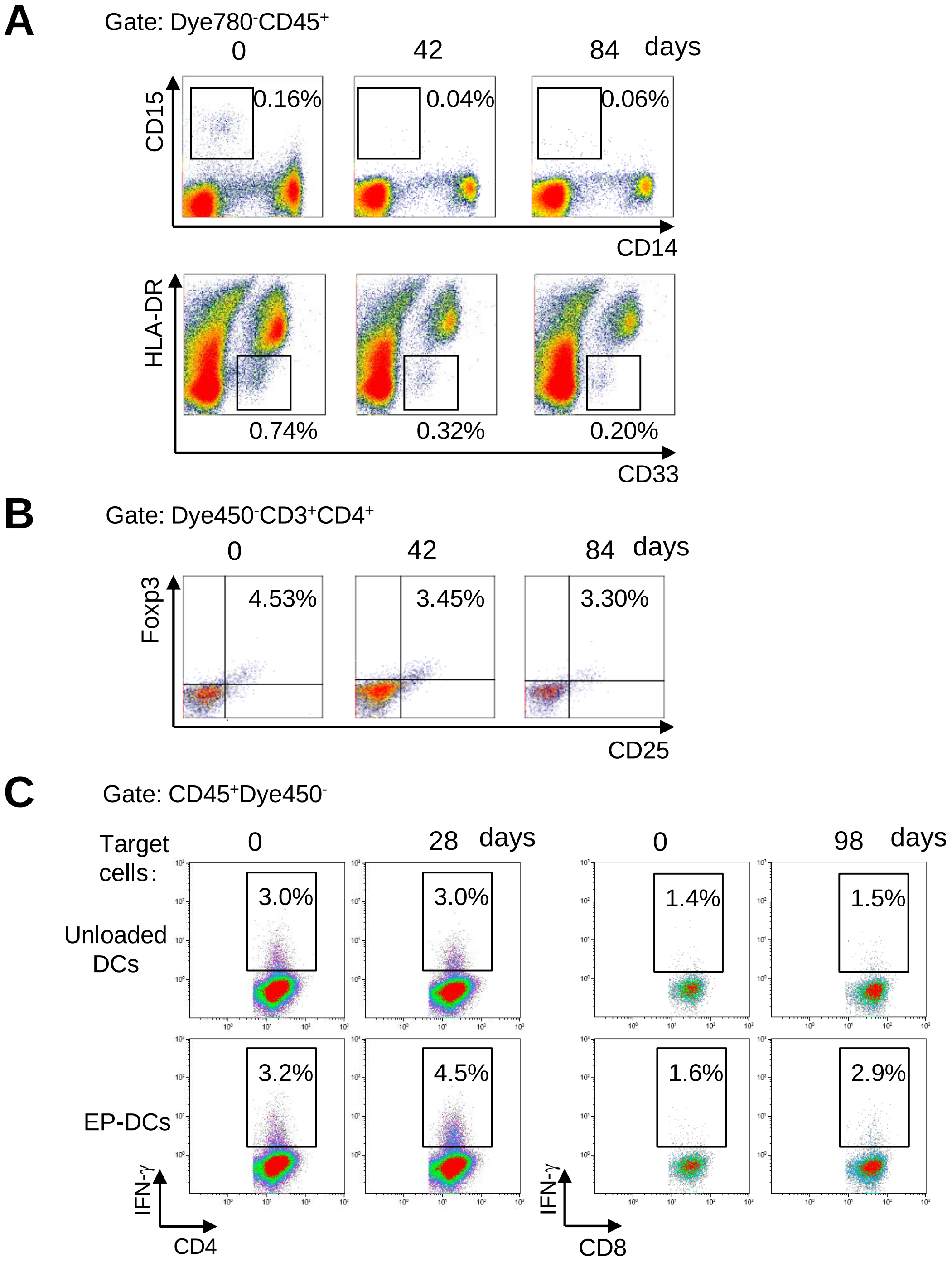

Additional File 3. Data from an individual patient. **A.** Data of frequencies of MDSCs over the course of treatment in patient #1802. The percentages of MDSCs in peripheral blood were evaluated by two criteria ($CD14^-CD15^+$ or $CD33^+HLA\text{-}DR^-$ as percentages of $Dye780^-CD45^+$ cells). **B.** Data of frequencies of Tregs in the course of treatment in patient #1814. The percentage of Tregs was evaluated as $CD25^+Foxp3^+$ cells within $Dye450^-CD3^+CD4^+$ cells. **C.** IFN-γ secretion assay for the detection of tumor-lysate reactive $CD4^+$ and $CD8^+$ T cells. Tumor-lysate reactive $CD4^+$ and $CD8^+$ T cell responses of patient #1802 before vaccination (day 0) and after 1st vaccination (day 28) or the 6th vaccination (day 98) are shown. PBMCs (1 x 10^6) from the patient before and after vaccination were stimulated once with EP-DCs (1 x 10^5) in the presence of IL-2 (50 U/ml) for 12 days. The cells (2 x 10^5) from the stimulation culture were assayed for IFN-γ secretion in response to EP-DCs (1 x 10^5) or unloaded DCs (1 x 10^5) for 4 hr using flow cytometry.
