# Supplementary material for: A pilot study of autologous tumor lysate-loaded dendritic cell vaccination combined with sunitinib for metastatic renal cell carcinoma
Source: J Immunother Cancer. 2014 Aug 19;2:30. doi: 10.1186/s40425-014-0030-4 (PMC4331924; doi:10.1186/s40425-014-0030-4)
Supplement: Additional file 4: — Computed tomography (CT) images. Supplementary figure. [file s40425-014-0030-4-S4.pdf]

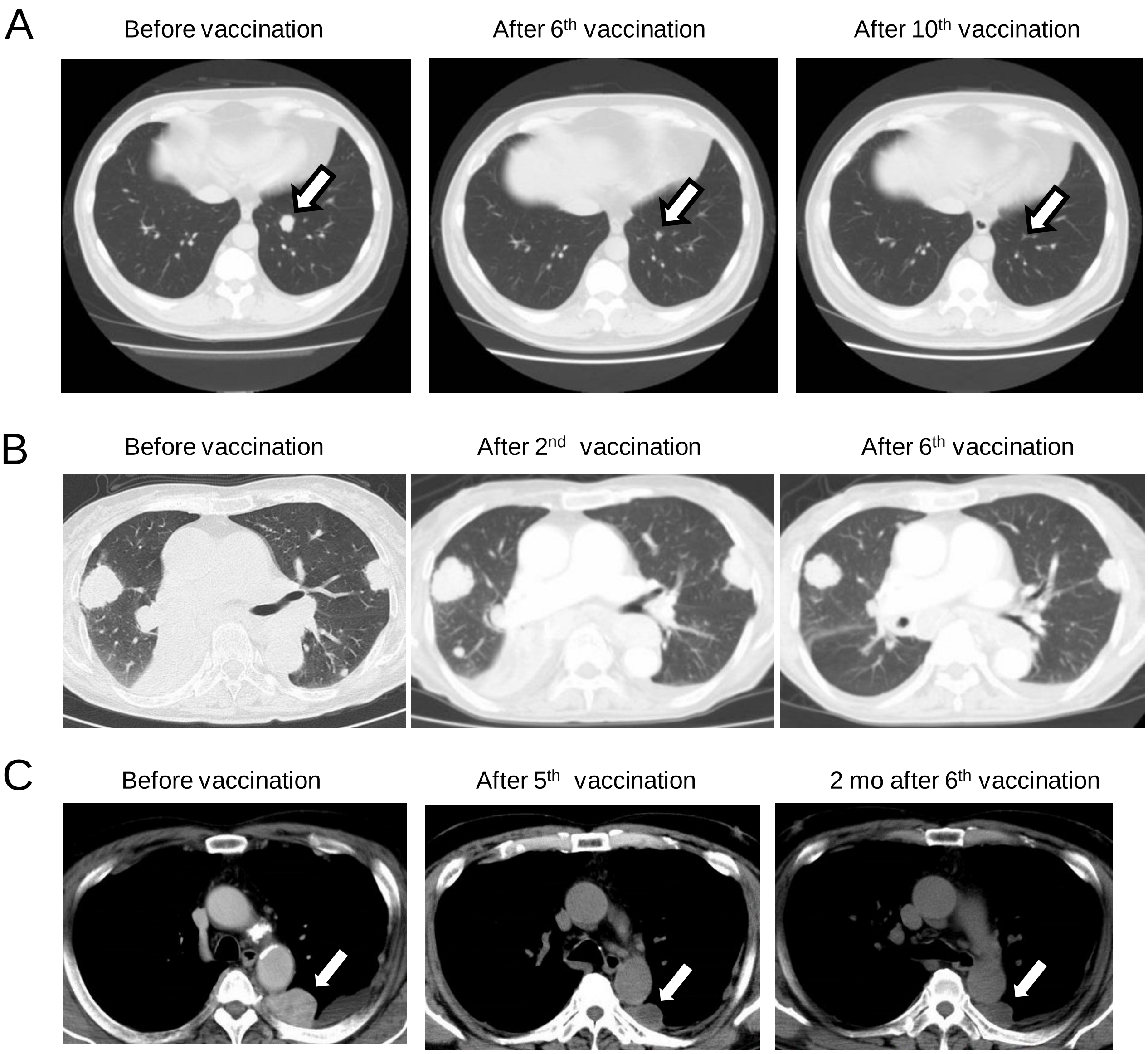

Additional File 4. Computed tomography (CT) images. **A.** CT images of patient #1814 showing the metastatic mass (arrow) in the left lung before vaccination, after the 6th vaccination and after the 10th vaccination. **B.** CT images of patient #1802 revealing multiple large masses in both lungs before vaccination, after the 2nd vaccination, and after the 6th vaccination. **C.** CT images of patient #1823 demonstrating reduction of tumor volume (arrow) during the course of the treatment.
